# Modern generative large language models in epilepsy care: a scoping review of current applications, challenges, and future directions

**DOI:** 10.1186/s42494-026-00271-5

**Published:** 2026-09-01

**Authors:** Shihao Ge, Yueqian Sun, Qun Wang

**Affiliations:** 1 Department of Neurology, Beijing Tiantan Hospital, Capital Medical University, No. 119 South 4th Ring West Road Fengtai District, Beijing, 100070 China; 2 Department of Neurology, The First Affiliated Hospital of Zhengzhou University, Zhengzhou, 450052 China; 3 National Center for Clinical Medicine of Neurological Diseases, Beijing, 100070 China

**Keywords:** Large language models, Generative artificial intelligence, Epilepsy, Clinical decision support, Natural language processing

## Abstract

Modern generative large language models (LLMs) are increasingly being evaluated in epilepsy-related clinical tasks, but the evidence remains fragmented and their safe clinical role is uncertain. We conducted a scoping review following the PRISMA-ScR framework, searching PubMed, Embase, and the Web of Science Core Collection from inception to May 2026, to map current applications of modern generative LLMs in epilepsy-related clinical contexts and identify their reported benefits, limitations, and research gaps. Eligible studies evaluated generative LLMs or generative foundation models as the primary analytical or assistive engine in clinical or clinically oriented epilepsy tasks; non-generative natural language processing, conventional machine-learning, and signal-modeling studies served as contextual comparators only. Two reviewers independently screened records and extracted data for descriptive mapping and narrative synthesis. Twenty-four studies were included. Evidence was relatively more developed for text-centered tasks, including information extraction from electronic health records, question answering, and documentation support, while applications in differential diagnosis, presurgical evaluation, prognostic assessment, and patient communication remained early-stage. Across studies, the evidence base was heterogeneous, with frequent reliance on retrospective or simulated designs, single-center data, and limited external validation, alongside persistent concerns about hallucination, bias, model opacity, and workflow integration. Current evidence suggests a limited role for cautious, clinician-supervised use of generative LLMs in selected text-heavy epilepsy tasks, particularly extraction, summarization, documentation support, and patient education, but falls short of justifying independent or routine clinical deployment. Future studies should use narrower task definitions, epilepsy-specific datasets, transparent reporting of model versions and prompt design, external validation, and explicit documentation of clinician verification workflows.

## Background

Epilepsy is one of the most common neurological disorders worldwide and substantially affects health and quality of life [1–4]. Although electroencephalography, neuroimaging, and other objective data are important in epilepsy care, many key clinical decisions still depend on accurate interpretation of unstructured narrative information, particularly patient history and seizure semiology [5, 6]. Despite widespread adoption of electronic health records (EHRs), much of this clinically valuable information remains embedded in free-text documents, including progress notes, imaging reports, and family descriptions of seizures [7]. Under routine time constraints, clinicians may find it difficult to extract key phenotypic information efficiently and consistently from these heterogeneous text sources. This limits both individualized clinical assessment and the secondary use of large-scale clinical data for epilepsy research [8]. Accordingly, better methods for structuring and using unstructured textual information remain an important need in epilepsy care and research.

In recent years, modern generative large language models (LLMs), exemplified by Generative Pre-trained Transformer (GPT) models, have emerged as a potentially useful technical approach for text-centered clinical tasks [9]. Through large-scale pretraining on diverse textual corpora, these models can support language understanding, summarization, information extraction, and question answering, which makes them relevant to several documentation- and review-intensive tasks in healthcare [10, 11]. Preliminary studies suggest that generative LLMs may have value in epilepsy-related knowledge tasks. For example, early evaluations have reported reasonable performance on neurology knowledge questions [12]. For epilepsy care, a plausible use is clinician-supervised extraction, organization, and summarization of narrative records, including patient chief complaints, electroencephalography (EEG) reports, and discharge summaries, to reduce manual review burden [13, 14]. 

However, the evidence base for generative LLMs in epilepsy remains early-stage and heterogeneous. Their effectiveness, safety, and appropriate role in real-world workflows are not yet well defined [10, 13]. Accordingly, this scoping review aims to map the current clinically oriented applications of modern generative LLMs in epilepsy and to distinguish relatively more developed text-centered use cases from less mature or higher-risk applications. The review therefore emphasizes evidence maturity, external validation, workflow fit, inaccurate or fabricated outputs, privacy, bias, and explainability, with the aim of separating plausible near-term uses from applications that remain too weakly validated for clinical reliance.

## Methods

This review was designed as a scoping review and reported according to the Preferred Reporting Items for Systematic reviews and Meta-Analyses extension for Scoping Reviews (PRISMA-ScR) framework. PubMed, Embase, and the Web of Science Core Collection were searched from inception to May 2026 for studies on epilepsy and modern generative large language models. The search strategy used two predefined concept blocks: epilepsy-related terms and generative LLM- or AI-chatbot-related terms. Records from the databases were deduplicated before screening. Two researchers independently screened titles/abstracts and then full texts against predefined eligibility criteria, and charted data using a structured extraction form that captured publication year, model type, task domain, data source, study design, evaluation setting, primary metrics, validation approach, and reported limitations; disagreements were resolved through discussion or consultation with a third senior researcher. Reasons for full-text exclusion were documented and summarized in the PRISMA-ScR flow diagram. No protocol was prospectively registered, and no separate grey-literature search was undertaken. Accordingly, the purpose was to map the available evidence, summarize application domains, and describe reported limitations rather than to perform formal evidence grading or risk-of-bias assessment. Original English-language research studies evaluating modern generative LLMs or generative foundation models in epilepsy-related clinical or clinically oriented tasks were eligible for descriptive mapping and narrative synthesis. Reviews, commentaries, conference abstracts, preprints, patents, studies without accessible full text, duplicate publications, and non-epilepsy studies were excluded.

For reproducibility, the core PubMed query was: ((epilep*[Title/Abstract]) OR (seizur*[Title/Abstract])) AND ((“large language model”[Title/Abstract]) OR (“large language models”[Title/Abstract]) OR LLM[Title/Abstract] OR ChatGPT[Title/Abstract] OR GPT[Title/Abstract] OR Claude[Title/Abstract] OR Gemini[Title/Abstract] OR Llama[Title/Abstract] OR Mistral[Title/Abstract] OR DeepSeek[Title/Abstract] OR Copilot[Title/Abstract] OR chatbot*[Title/Abstract] OR (“foundation model”[Title/Abstract]) OR (“foundation models”[Title/Abstract])). The corresponding Web of Science topic-field query was: TS=(epilep* OR seizur*) AND TS=(“large language model*” OR LLM OR ChatGPT OR GPT OR Claude OR Gemini OR Llama OR Mistral OR DeepSeek OR Copilot OR chatbot* OR “foundation model*”). An equivalent Embase strategy combined Emtree and title/abstract/keyword terms for epilepsy or seizures with generative LLM- and AI-chatbot-related terms. Searches were last updated in May 2026.

A key inclusion criterion was that a modern generative LLM or generative foundation model served as the primary analytical, reasoning, or assistive engine in an epilepsy-specific clinical, patient-facing, or clinically oriented research task. Because the term “LLM” is used inconsistently in the recent literature, this review adopted an operational definition that restricted the main synthesis to studies centered on modern generative, instruction-following, or free-text-generating models [15, 16]. Encoder-style clinical natural language processing (NLP) models, conventional machine-learning classifiers, and seizure-prediction signal-modeling frameworks were not treated as core generative-LLM evidence. If such literature was cited, it was used only to explain the broader epilepsy-AI context, methodological comparators, or translational boundary conditions, and was not counted in the core synthesis.

## Results

**Fig. 1:**
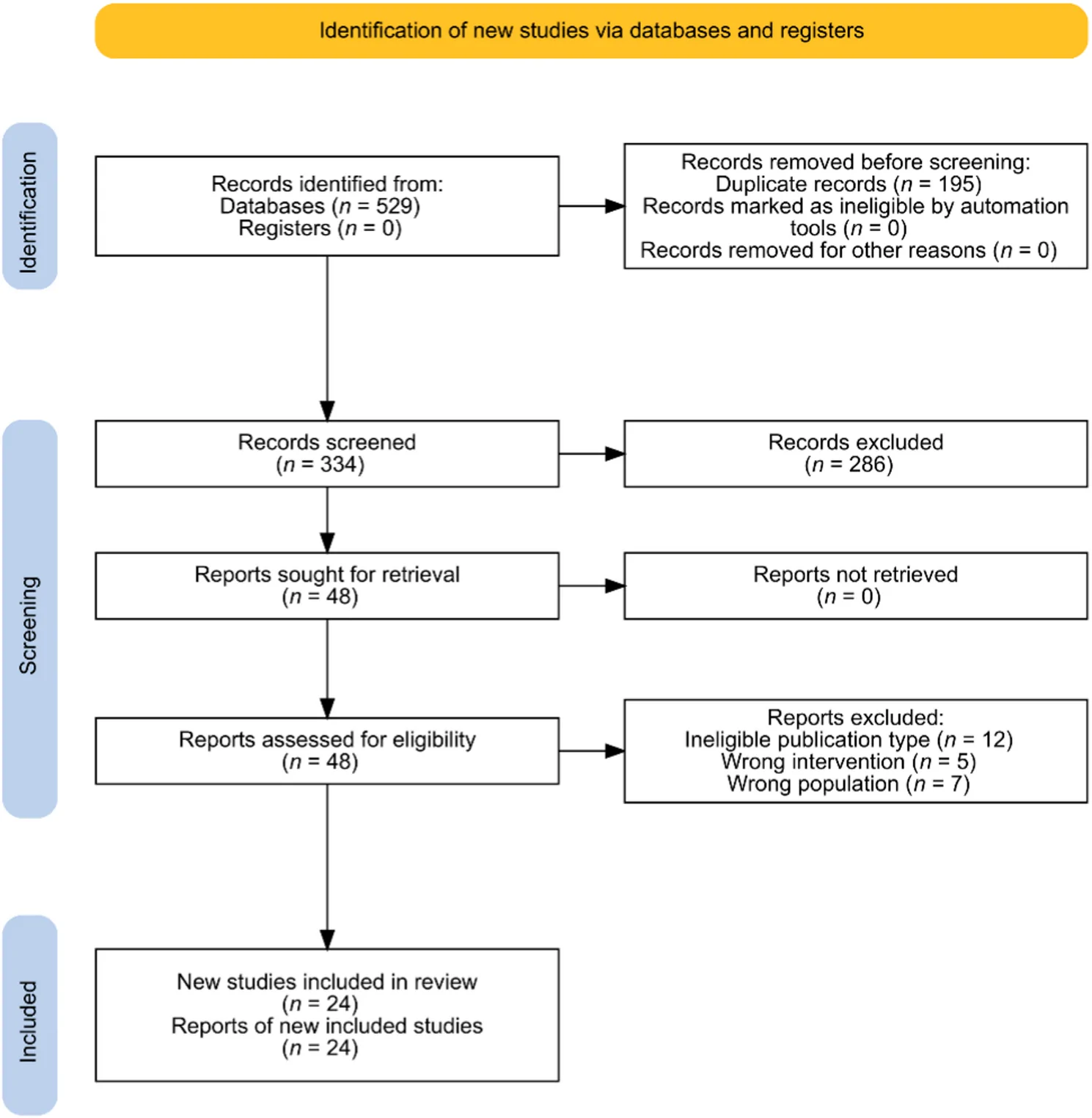
PRISMA-ScR flow diagram of the study selection process

The database search identified 529 records across PubMed, Embase, and Web of Science, of which 334 remained after removal of 195 duplicates. After title/abstract screening, full-text assessment, and manual rechecking against the eligibility criteria, 24 studies were retained for descriptive mapping and narrative synthesis; their main characteristics are summarized in Table 1. The study selection process is summarized in the PRISMA-ScR flow diagram (Fig. 1) [17]. 

**Table 1:** Basic characteristics of included studies

Reference	Study focus	Model(s)	Evaluation setting	Main finding(s)	Limitations / note
**Clinical diagnosis**					
Chiang et al. [18]	Support for epilepsy surgery localization.	Customized GPT/JSON matcher with ontology-based retrieval augmentation	Retrospective surgical sample of 16 operated cases.	With symptoms alone/plus EEG, accuracy was 75%/87.5% for the ontology system and 87.5%/93.8% for the JSON matcher.	Very small selected cohort; retrospective text matching rather than prospective decision support.
Brigo et al. [19]	Diagnosis of epilepsy using the International League Against Epilepsy (ILAE) practical definition in emergency-department cases.	ChatGPT-4	Single-center retrospective emergency-department cohort of 597 patients.	ChatGPT-4 diagnosed epilepsy less often than neurologists; sensitivity was 17.6% and specificity was 81.4%.	Poor sensitivity and absent agreement make this approach unsuitable for diagnostic support in its current form.
Brigo et al. [20]	Adult seizure and epilepsy diagnosis/classification.	ChatGPT-4	Prospective simulation using 37 adult clinical vignettes.	Sensitivity was high for seizure/epilepsy identification, but specificity for epilepsy was low at 26.7%.	Small vignette study with frequent false positives; findings support only adjunctive evaluation.
Ford et al. [21]	Classification of epileptic versus functional seizures from patient descriptions.	GPT-4	41 transcript cases evaluated with zero-shot and few-shot prompting.	Balanced accuracy improved from 57% to 64% with one-shot prompting but remained below neurologists at 71%.	Small sample without uniform video-EEG confirmation; performance depended on text detail and remained below specialists.
Kerr et al. [22]	Identification of functional seizures from medical-record vignettes.	ChatGPT-3.5, GPT-4	114 de-identified cases tested with sequential clinical information and repeated prompts.	Under modified referral-oriented scoring, GPT-4 reached 85% accuracy and an area under the receiver operating characteristic curve (AUC) of 87%; predictions changed across days in 33% of patients.	Good headline performance was limited by output instability and only fair agreement with structured scores.
Patel et al. [23]	Video-only classification of epileptic versus functional seizures.	Gemini multimodal models	24 smartphone videos from 15 patients, with video-EEG reference and no clinical context.	Accuracy remained modest at 25.0–54.2% despite high self-rated confidence.	Very small imbalanced sample; video quality and framing limited autonomous classification.
Luo et al. [24]	Semiology-based localization of the epileptogenic zone.	ChatGPT-4	852 published semiology-localization pairs plus 184 private cases.	Sensitivity was approximately 80–90% for frontal and temporal zones but much lower for rarer regions.	Semiology-only task; possible public-case overlap and weak performance for rare localizations.
Tan et al. [25]	Triage and chart collation for epilepsy surgery candidacy.	Mistral 7B Instruct	Single-site 12-month implementation; top 5% of 310 screened patients underwent manual review.	Eight of 15 high-risk cases met surgery-evaluation criteria, with extraction accuracy of 80–100%.	Only 15 cases were manually reviewed; hallucination, omission, and copy-forward errors required human checking.
Fennig et al. [26]	Routine-note screening for surgery eligibility.	Gemini models, GPT-5, GPT-5 mini, o4-mini	Retrospective Hebrew clinical-note study of 110 patients with expert review as reference.	Majority voting achieved sensitivity 1.00 and specificity 0.96; 13 of 29 eligible patients had not previously been considered for surgery.	Single-center non-English data; prompt refinement and prospective workflow testing remain necessary.
Sun et al. [27]	Presurgical epileptogenic-zone localization from text reports.	GPT-4.1, DeepSeek-R1, Claude 3.7	Two-center retrospective study of 154 seizure-free surgical cases.	Laterality accuracy was 98.3–100%, but lobar and stereoelectroencephalography-related performance varied by model and input source.	Enriched seizure-free cohort using text reports only; generalizability to broader presurgical workups is limited.
**Extract structured information**					
Abeysinghe et al. [28]	Extraction of seizure frequency from epilepsy-monitoring-unit reports.	Fine-tuned GPT-4; GPT-3.5; Llama-2; BERT comparators	Single-dataset supervised extraction benchmark.	Fine-tuned GPT-4 achieved structured-output F1 score of 85.82%.	Bespoke epilepsy monitoring unit report workflow with segment-level evaluation; external validation is needed.
Fang et al. [29]	Extraction of epilepsy information from clinic letters.	Llama/Mistral/Mixtral open-source models	280 annotated clinic letters compared with human labels and MedCAT.	The best model achieved F1 of 0.80 for epilepsy type, 0.76 for seizure type, and 0.90 for current antiseizure medications (ASMs).	Single UK-center dataset; implicit or specialized information remained difficult and outputs were not autonomous.
Fennig et al. [30]	Detection of patient concerns and mental-health signals in Reddit posts.	ChatGPT-4-Turbo, ChatGPT-4o	56,970 r/epilepsy posts with cross-subreddit activity analysis.	The models identified themes associated with later depression/suicidality-forum posting; epilepsy and monthly-seizure discrimination AUCs were 0.79 and 0.77.	LLM topic fidelity was low; Reddit users are nonrepresentative and results should not be used for individual prediction.
Goldenholz et al. [31]	Abstraction of synthetic trial notes for treatment-signal estimation.	Llama-2, Mistral, Claude	Fully simulated cenobamate trial notes from 240 synthetic patients.	AI-derived efficacy estimates differed from human review by 1%, and symptom rates by at most 3%.	Synthetic-note proof of concept only; real trial abstraction was not validated.
Golnari et al. [32]	Ontology-guided extraction of antiseizure-drug evidence in Dravet syndrome.	Gemini 1.0 Pro with ontology-augmented prompting	Expert benchmark of 17 ASMs; 4,935 articles screened to a 75-article validation corpus.	Ontology-augmented prompting reproduced the expert benchmark with 100% accuracy using two examples.	Performance depended on an expert-built ontology and benchmark; this was literature extraction, not clinical validation.
Ojemann et al. [33]	Zero- or few-shot seizure-outcome extraction from clinical notes.	Llama-2-13B with BERT comparators	Annotated single-center note corpus evaluated against fine-tuned BERT models.	The best prompt achieved 74–76% accuracy across epileptologist and generalist notes.	Temporal reasoning remained difficult and performance was inferior to fine-tuned BERT on specialist notes.
Kamalmaz et al. [34]	Surgery outcome extraction for cost-effectiveness analysis.	Mistral 24B pipeline	Single-center retrospective surgical EHR study compared with a prospective database.	Engel outcome extraction accuracy was 95.65%; good-versus-bad outcome accuracy was 91.2%, and incremental cost-effectiveness ratio (ICER) differed by 2.08%.	Only 69 surgical cases were used for LLM comparison; results depended on documentation quality and do not establish economic decision-making.
**Medical advice**					
Wu et al. [35]	Responses to common epilepsy questions and emotional-support prompts.	ChatGPT-3.5	378 curated epilepsy questions plus five emotional-support prompts graded by epileptologists.	Responses were correct and comprehensive in 68.4% of cases and reproducible in 82.3%; prognosis answers were weakest.	Curated questions and an older model were used; incomplete or unsafe answers remained possible.
Jesus-Ribeiro et al. [36]	Responses to Dravet syndrome questions from professionals and caregivers.	ChatGPT-3.5, Perplexity	96 curated Dravet syndrome questions rated for accuracy, completeness, and readability.	Correct-answer rates were 81.3% for Perplexity and 66.7% for ChatGPT; treatment questions were weakest.	Curated benchmark with advanced reading level; treatment guidance still requires expert review.
Rocha-Silva et al. [37]	Quality and readability of epilepsy/exercise information.	ChatGPT-3.5/4, Gemini, Copilot	14 exercise-related questions evaluated by seven blinded expert raters.	Responses were generally acceptable; ChatGPT-4 was most often preferred and Copilot was most readable.	Only 14 questions were tested; readability remained difficult and some advice was inconsistent with evidence.
Ajmera et al. [38]	Therapeutic ketogenic-diet planning for children with epilepsy.	ChatGPT, Copilot, Gemini	Three pediatric ketogenic-diet scenarios evaluated by dietitians.	All chatbots failed to generate safe or accurate diet plans, with major ratio and food-suitability errors.	Although vignette based, clinically actionable errors show that specialist oversight is essential.
Birkun et al. [39]	First-aid counselling during generalized seizure scenarios.	Baseline and customized GPT-4o	Four scripted bystander/postictal scenarios across 120 dialogues.	Customization improved checklist fulfillment from 14–49% to 77–92%.	Scripted text-only scenarios were used; omissions persisted and real bystander settings were not tested.
Kliem et al. [40]	Status-epilepticus guideline adherence.	ChatGPT-3.5, GPT-4o, GPT-5	Repeated hypothetical status-epilepticus prompts across freely available ChatGPT versions.	More detailed prompts improved guideline adherence, but side positioning, dosing, assessment, and complications were often missed.	Prompt- and model-sensitive outputs remain unsafe for unverified treatment guidance.
**Prognostic prediction**					
Amacher et al. [41]	Outcome prediction after status epilepticus.	ChatGPT-4o	Single-center retrospective cohort of 760 adult status-epilepticus patients compared with the Status Epilepticus Severity Score (STESS).	The model had limited utility, with survival sensitivity of 75.8% and specificity of 36.6%, and no advantage over STESS.	Restricted inputs, weak discrimination, and black-box reasoning limit bedside prognostication.

### Adjunctive and differential-diagnostic tasks

Evidence for generative LLMs in diagnostic and differential-diagnostic tasks remains preliminary and task-dependent. Clinically grounded evaluations showed substantial limitations. In a retrospective emergency-department study applying the 2014 ILAE practical definition, ChatGPT-4 diagnosed epilepsy less often than neurologists and showed essentially absent agreement, with very low sensitivity despite moderate specificity [19]. In a prospective simulation of 37 adult vignettes derived from real seizure presentations, ChatGPT-4 showed high sensitivity for identifying epileptic seizures and diagnosing epilepsy (≥ 96.9%) but poor specificity for diagnosing epilepsy (26.7%) and distinguishing acute symptomatic from unprovoked seizures (33.3%); human experts consistently performed better [20]. These findings do not support stand-alone diagnosis, and any adjunctive role requires further clinical evaluation.

Evidence for distinguishing epileptic from functional or dissociative seizures is also preliminary and sensitive to task framing. On patient narratives, GPT-4’s balanced accuracy improved from 57% in the zero-shot setting to 64% with one-shot prompting, without further gains from additional examples. Performance remained below that of experienced neurologists (71%) and reached 81% only in the 18 cases all neurologists classified correctly [21]. In another study, GPT-4 received information sequentially: history, EEG, and then neuroimaging. The authors also reported a modified, referral-oriented analysis in which a prediction of both epileptic seizures (ES) and functional seizures (FS) for a patient with FS alone was treated as clinically relevant because it still identified possible FS and could prompt further evaluation. Under this analysis, GPT-4 achieved 85% accuracy and an AUC of 87%, outperforming ChatGPT and a supervised functional-seizure score [22]. However, agreement with the structured score was only fair, and repeated runs changed predictions in 33% of patients. Thus, favorable discrimination did not translate into stable decisions, and the added value over structured tools remains uncertain.

A small prospective multimodal study examined 24 smartphone-recorded events from 15 patients at a tertiary center. Without additional clinical context, Gemini models achieved only 25.0–54.2% accuracy in distinguishing epileptic from functional seizures, despite high self-rated confidence and somewhat better performance in upper-body or face-focused recordings [23]. This video-only task does not directly inform text-based diagnosis, but it shows that current multimodal LLMs cannot reliably classify patient-recorded events without clinical context.

Presurgical evidence differed primarily in how much clinical information had already been organized before reaching the model. In 16 single-center surgical cases, investigators converted an expert-built epilepsy ontology into a JSON knowledge base for a customized GPT matcher using retrieval-augmented generation (RAG). Adding EEG text improved accuracy from 75.0% to 87.5% for the ontology system and from 87.5% to 93.8% for the GPT/JSON matcher; because the integrated system was evaluated without ablation, the improvement cannot be attributed to RAG alone [18]. With semiology alone, ChatGPT-4 showed similar patterns in public and private cohorts but remained sensitive to case representation: regional sensitivity was 80–90% for frontal and temporal localizations, 20–40% for parietal, occipital, and insular regions, and 3% for the cingulate cortex. Because semiology reflects the symptomatogenic zone, these predictions did not necessarily identify the epileptogenic zone itself [24]. Multisource specialist reports yielded 98.3–100% laterality accuracy in 154 seizure-free surgical patients from two centers, yet lobar performance remained model- and subtype-dependent; medical records and magnetic resonance imaging reports contributed most in ablation analysis, and insular localization remained poorest [27]. Together, these studies distinguish evidence integration from independent localization: performance was strongest for broad or well-represented targets supported by informative text, whereas rare and anatomically specific targets remained difficult.

### Structured information extraction from unstructured text

Evidence for generative LLMs is currently most consistent in narrowly specified extraction tasks from narrative clinical text. Seizure-frequency extraction is a good example: a fine-tuned GPT-4 pipeline achieved an F1 score of 85.8% for structured seizure-frequency output and outperformed several BERT-based and generative comparator models [28]. A separate clinic-letter study similarly found that open-source LLMs could extract epilepsy type, seizure type, current antiseizure medications, and associated symptoms more effectively than a conventional clinical NLP comparator, although implicit and specialized information remained more difficult [29]. However, these performances were obtained within carefully defined extraction workflows and should be interpreted as evidence for task-bounded information structuring rather than broader clinical reasoning. Zero-shot or prompt-engineered approaches may still offer practical value when annotation resources are limited. In seizure-outcome extraction, prompt-engineered Llama2 reached 74% median accuracy on epileptologist notes, outperforming zero-shot BERT (25%) but remaining below the fully fine-tuned BERT benchmark (84%); in sparser general-neurology notes, the best prompt reached 76% and exceeded the fine-tuned BERT model (67%) [33]. A 2026 single-center cost-effectiveness study extended this extraction paradigm to real-world surgical-outcome surveillance: a Mistral 24B pipeline extracted Engel outcomes from EHR notes with 95.65% extraction accuracy, supported 91.2% binary good-vs-bad outcome classification, and generated an ICER estimate that differed by only 2.08% from the prospective-database approach [34]. These studies show task-specific feasibility for text extraction but do not establish safe unsupervised clinical use.

Off-the-shelf generative LLMs have also been tested for clinician-facing triage based on routine notes. In a retrospective tertiary-clinic study using raw Hebrew clinical notes from 110 patients, GPT-5, GPT-5 mini, o4-mini, and Gemini models identified epilepsy-surgery eligibility with sensitivity up to 1.00 and specificity up to 0.96; majority voting captured all eligible patients in that cohort. Among patients who met surgical criteria, 45% had not previously been considered for surgery. This result supports further evaluation of LLM-based referral flagging under clinician review. The single-center retrospective design does not support autonomous surgical triage [26]. 

Implementation-oriented evidence remains sparse, but one recent single-site study moved closer to a realistic workflow by combining conventional triage with zero-shot generative extraction. Over a 12-month period, a previously developed random-forest screen was used to flag the top 5% of 310 clinic patients for manual review, and a Mistral-7B-Instruct model was then used to collate referral-relevant information from the chart. Among the 15 manually reviewed high-risk cases, 8 met prespecified criteria for epilepsy-surgery evaluation and only 3 were referred within 1 month. However, although extraction accuracy for selected structured elements ranged from 80% to 100%, performance was weaker for management-plan summarization and the LLM produced hallucination, omission, and copy-forward errors [25]. This implementation-style study therefore supports a narrow role for LLMs in surfacing referral-relevant text for specialist review, not autonomous referral decision-making.

### Epilepsy prediction and disease prognosis

Evidence for prognosis remains limited to one included study. In a single-center retrospective cohort of adults with status epilepticus, ChatGPT-4o achieved sensitivity of 75.8% but specificity of only 36.6% for discharge survival, showed no advantage over STESS, and produced unstable results across two runs of the same standardized prompt [41]. Current evidence therefore does not justify general-purpose LLMs as stand-alone bedside prognostic tools.

### Patient education and physician-patient communication

A 2026 comparative study tested ketogenic diet planning for children with epilepsy, a task requiring precise and clinically actionable dietary calculations. ChatGPT, Copilot, and Gemini all produced unsafe or inaccurate menus, with major errors in macronutrient targets, ketogenic ratios, and food suitability across all tested scenarios, including classic ketogenic, medium-chain triglyceride, and modified Atkins diets. The models’ fluent outputs still contained clinically important errors when specialist dietary precision was required [38]. 

LLMs show preliminary potential as sources of epilepsy-related health information, but accuracy, readability, and reliability remain limiting. In an earlier benchmark of common epilepsy questions and emotional-support prompts, ChatGPT-3.5 produced correct and comprehensive responses in only about two-thirds of cases, with prognosis-related answers performing particularly poorly [35]. In curated patient-information benchmarks, ChatGPT-4 was generally rated favorably for epilepsy-and-exercise questions, and Perplexity achieved 81.3% accuracy on Dravet syndrome questions. Nevertheless, treatment-related Dravet questions remained the weakest area, expert review identified evidence-inconsistent exercise advice, and clinically important errors persisted despite favorable overall performance [36, 37]. In emergency-oriented settings, the risk was more direct: ChatGPT recognized benzodiazepines as first-line therapy for status epilepticus but omitted or inconsistently reported several guideline elements unless prompts were made more specific, and misleading recommendations were still observed [40]. A seizure-first-aid counselling experiment similarly found that baseline GPT-4o failed to assess whether seizures were ongoing and whether the person was conscious or breathing, whereas an expert-customized GPT-4o improved checklist fulfillment from 14 to 49% to 77–92% but still required simulation and human validation [39]. LLMs may therefore be supplementary, clinician-supervised information tools, but direct patient-facing use remains unsafe without rigorous validation, appropriate readability, and specialist oversight.

LLMs may also be used to analyze large-scale, unstructured patient-generated data. In a study of the epilepsy community on Reddit, 56,970 posts from 21,906 users, together with their cross-subreddit activity, were analyzed to identify themes associated with later posting in depression- and suicidality-related forums. The study also compared LLM topic labels with clinician-reviewed human labels. Label fidelity was limited: the model recovered only 34% of human-identified topics, and only 18% of LLM-labeled topics matched human labels, although AUCs for identifying whether a poster likely had epilepsy and whether seizures had occurred in the previous month were 0.79 and 0.77, respectively [30]. This approach may support exploratory community-level analysis but not individual clinical risk prediction.

### Basic research and new paradigm exploration

Structured external knowledge may compensate for limited domain knowledge in specialized epilepsy tasks. In a Dravet syndrome study, investigators integrated an expert-built epilepsy ontology into a phased in-context learning pipeline for Gemini 1.0 Pro to extract drug-efficacy data from the literature. The ontology-augmented approach reproduced an expert benchmark covering 17 antiseizure medications with 100% accuracy using two in-context examples; 4,935 articles were screened to create a 75-article validation corpus [32]. The benchmark result came from a highly curated domain and required substantial expert modeling. Generalizability to other epilepsy subtypes and routine clinical tasks remains uncertain.

A separate proof-of-concept tested whether general LLMs could recover structured data from noisy text under controlled conditions. Investigators constructed a simulated randomized trial of cenobamate using synthetic seizure diaries and LLM-generated clinical notes, then used a multi-LLM pipeline to recover efficacy and safety signals. Compared with human review, the efficacy estimate differed by 1% and reported symptom rates by at most 3%. The system used general foundation models without medical fine-tuning [31]. Given the synthetic-record design, this result establishes feasibility only for controlled trial abstraction and does not support real-world clinical-note analysis or prospective trial workflows.

## Discussion

### Challenges and limitations

#### Inadequate performance in professional domains

Although LLMs show promise in processing clinical text, their use in diagnosis, treatment support, and documentation remains constrained by reliability and factual accuracy, with direct implications for patient safety. A recurring concern across epilepsy-focused commentaries and implementation reports is hallucination, whereby models may generate plausible but incorrect clinical content [42]. Published reports include unsafe medication advice, inappropriate surgery suggestions, and documentation errors such as confused drug names or altered dosages, all of which would be unacceptable without clinician review in high-stakes workflows [10, 43, 44]. 

This unreliability partly reflects the mismatch between general-purpose pretraining and highly specialized epilepsy tasks. Performance declines in rare syndromes, nuanced differential diagnoses, and other domain-specific scenarios, where knowledge gaps become more apparent [21, 32, 36]. Model outputs can also vary across repeated runs. In one comparison study, the same case yielded different predictions on different days in roughly one-third of patients, and the model’s self-rated certainty only partly tracked this variability [22]. Such run-to-run variability and incomplete self-calibration limit unsupervised clinical use.

#### Deficient clinical reasoning ability

LLMs can perform well on some neurology knowledge tasks [12]. This performance does not ensure reliable clinical reasoning. This limitation is especially evident in clinical hierarchy and process. One study showed that although LLMs could recognize well-defined syndromes, they often failed at the more basic step of diagnosing epilepsy before attempting syndrome classification [20]. Limitations become even more apparent in complex cases that require integration of multidimensional information. Even advanced models such as GPT-4 performed substantially worse than human experts when cases involved complicated medical histories or structural etiologies [19]. Overall, current LLMs remain more useful for bounded retrieval and pattern recognition than for expert-level clinical reasoning.

#### Data and knowledge dependency dilemma

Despite strong natural-language capability, epilepsy-related performance remains constrained by outdated or incomplete knowledge, training-data bias, and limited generalizability across tasks and settings. Most current studies evaluate retrospective record review, simulated reasoning, or educational responses rather than real-time, source-grounded retrieval and synthesis of current guidelines and research updates. This limited evidence base matters because fluent outputs can obscure whether claims are current, source-grounded, and clinically verifiable [44]. For epilepsy care, this creates a practical conflict with traceability and verification.

Sociodemographic bias is another major challenge to equitable clinical use. Existing research indicates that LLMs may learn and amplify biases related to race, gender, or socioeconomic status from their training data, potentially producing lower accuracy or unfair recommendations for underrepresented groups [11, 42, 44]. Until fairness and transparency are better validated in epilepsy-specific clinical settings, implementation may reproduce existing disparities in care.

Performance in epilepsy also remains highly task-dependent, and results from general-purpose LLMs should not be assumed to transfer directly to specialized scenarios. This contrast is already evident in the literature: seizure-description diagnosis with GPT-4 improved only modestly with prompting, whereas stronger seizure-frequency extraction depended on task-specific fine-tuning [21, 28]. Other retrospective epilepsy NLP studies likewise suggest that documentation style, local workflow, and institutional context can materially shape downstream performance [45]. Reliable clinical use will therefore require high-quality annotation, external validation, and caution against treating any single proof-of-concept task as evidence of broad epilepsy competence.

#### Clinical integration and barriers to application

Even when epilepsy-focused studies report promising accuracy, clinical translation remains limited by opacity, instability, and governance barriers. High-performing models may generate useful predictions from routine notes while still offering reasoning that is difficult to inspect [46, 47]. Many widely used systems are commercial or cloud-mediated, raising privacy, local-deployment, and institutional-governance concerns for identifiable clinical text [11, 14]. These factors help explain why promising retrospective accuracy has not yet translated into dependable clinical integration.

Evaluation methods and unresolved ethical and legal issues continue to limit clinical adoption. Medical LLM studies vary in tasks, prompts, raters, and outcome metrics, limiting cross-study comparison and assessment of clinical relevance [14]. For epilepsy-related tasks, accuracy or AUC should be accompanied by calibration, reproducibility, unsafe-error analysis, and the clinician effort needed to identify and correct errors. Liability for model-related harm remains unresolved [44]. Taken together, the current evidence favors supervised research support, population-level pattern analysis, and structured knowledge extraction over autonomous treatment guidance [31]. Patient-facing outputs may also be difficult for nonexperts to interpret safely, even when rated as accurate [36]. Clinical interpretation therefore depends on explicit oversight and transparent reporting.

Accordingly, clinically oriented evaluations should report core study metadata, including model version, evaluation date, prompt design, and repetition strategy. They should also describe the data source, annotation procedure, rater expertise, uncertainty handling, and prespecified adjudication of unsafe outputs. These details are needed to judge reproducibility, source fidelity, clinician correction burden, and the consequences of false reassurance or over-referral, which headline accuracy or AUC alone cannot capture. Explainability should also be interpreted differently across model families: signal-based EEG classifiers may sometimes be interrogated with saliency maps, SHAP-style feature attribution, or class-activation approaches, whereas LLM explanations require source-grounded reasoning, traceable links to the clinical record, and pathophysiological plausibility rather than fluent post hoc rationales alone.

These concerns are amplified in rare-event monitoring or prognostic tasks, where class imbalance and false-alarm burden can make high AUC or sensitivity misleading. Cost-sensitive EEG classifiers illustrate one way to encode unequal false-negative and false-positive costs under severe imbalance, but they should be treated as methodological comparators rather than generative-LLM evidence [48]. In such settings, clinically meaningful evaluation must go beyond headline discrimination to include precision-recall behavior, false-positive workload (including false alarms per hour where applicable), detection latency, and calibration [49–52]. Future LLM studies that make monitoring, triage, or prognostic claims should therefore prespecify clinically meaningful escalation thresholds and report false-alarm rates and calibration, rather than relying on aggregate discrimination metrics alone. This limitation reflects a mismatch between the current evidence base and the requirements of real-time detection: included generative-LLM studies mainly used text or episodic inputs, whereas continuous seizure monitoring requires temporally localized signal processing with predictable latency, artifact robustness, and prospectively validated false-alarm burden. At present, this evidence does not support real-time seizure detection or autonomous early-warning deployment by generative LLMs.

### Outlook

#### Building an epilepsy-specific evidence base

The performance of general-purpose models depends on the completeness and clinical relevance of the information provided. More detailed patient narratives were associated with better discrimination [21]. By contrast, additional EEG or imaging information did not consistently improve functional-seizure classification [22]. Different clinical reports also provided unequal value for presurgical localization [27]. Across these tasks, additional information was useful when it was relevant to the specific clinical question, not simply because more data were provided. To support this, future model development will require well-curated, expert-annotated epilepsy datasets that include rare and ambiguous cases from multiple centers. External validation should then establish whether the resulting models remain reliable outside the development setting.

Improving epilepsy applications will require matching the adaptation strategy to the task. Structured prompting and expert-curated content have improved first-aid and guideline-based tasks [39, 40], ontology guidance and ontology-based RAG have been evaluated for terminology standardization, evidence extraction, and presurgical localization [18, 32], and fine-tuning has benefited narrow, repeated extraction [28]. These approaches address different limitations, so the adaptation strategy should be selected according to the task; no single method should be assumed to be universally superior. RAG has so far been examined only within one small integrated presurgical system, while another diagnostic study proposed it as future work rather than testing it [18, 21]. Future studies should compare prompting, fine-tuning, ontology guidance, and RAG on the same datasets and outcomes, and assess source accuracy, currency, clinically important errors, and external validity.

Multimodal development should determine whether each modality improves a specific clinical decision rather than assume that more input is always better. Current epilepsy studies have largely relied on clinical histories and written EEG and imaging reports [18, 27]. Direct analysis of raw EEG, imaging, or video data remains largely unexplored; the available video study was exploratory and showed poor performance, particularly when recording quality or framing was inadequate [23]. Future work could therefore test whether raw EEG, imaging, or video adds information beyond text reports. Where direct multimodal interpretation remains unreliable, one possible approach would be to combine LLMs with established signal- or image-analysis models and pass their structured outputs to the language model. Such hybrid approaches would still require task-specific evaluation of incremental value, noise, and input quality, together with an abstention mechanism for cases in which the available evidence is insufficient.

#### Clinician-overseen and auditable implementation

Clinical implementation is easier to audit when model outputs remain traceable to their source records. This is possible in extraction workflows, where variables can be checked against the record [28, 29], and in referral screening, where specialists can adjudicate flagged cases [25, 26]. By contrast, current diagnostic, classification, and prognostic applications convert conflicting or incomplete evidence into patient-level judgments [19, 20, 22, 41], making it harder to trace an error to a specific source element. LLMs should therefore organize evidence for clinical review, preserve links to source records, and indicate when evidence is conflicting or incomplete, rather than provide a final clinical judgment.

Future implementation studies should determine whether LLM-assisted screening improves care, rather than report model accuracy alone. For periodic re-screening of patients who may have missed surgical evaluation, studies should distinguish process measures, including newly identified candidates, clinical review burden, and completed referrals, from patient outcomes [25, 26]. Because output variability [22, 26, 41], overconfidence [23], and missing source links [25] may affect which patients are flagged and why, studies should also report repeatability and source traceability. Implementation studies should also retain an auditable record of model outputs, clinician corrections, and identified errors.

Local or offline deployment may be useful when workflows repeatedly process identifiable records or require a fixed model version. Keeping data within hospital infrastructure can strengthen privacy protection [26, 29]. Institutional control over the model and prompts may improve reproducibility across screening cycles. However, direct comparisons with cloud systems are lacking. Local deployment also requires suitable hardware and may restrict model choice. In one study, hardware limitations constrained the model size that could be used [33].

#### Research and population-level applications

When LLM-extracted variables are used to construct cohorts, define outcomes [28, 29, 33], or inform health-economic models [34], it is not enough to verify individual extracted variables in isolation. Extraction errors can alter economic estimates [34]. They may also cause uncommon adverse events to be missed even when aggregate results appear accurate [31]. Future pipelines should combine automated extraction with manual checks that deliberately include difficult and rare cases, and use sensitivity analyses to test whether plausible extraction errors change effect estimates, subgroup findings, or safety conclusions.

Beyond record-based research, future work could use LLMs to integrate concerns expressed during routine consultations, follow-up, patient-reported outcomes, and online communities, helping identify needs missed by structured records. Current epilepsy-specific evidence remains limited to exploratory analysis of patient-generated online data [30], which may reflect selective contributors and yield themes that do not fully agree with human interpretation. Future studies should test whether combining these signals with longitudinal clinical records can support population-level assessment of psychosocial burden and unmet support needs, rather than infer prevalence or individual risk from online data alone; any use in personalized follow-up or service planning should be validated with patients, clinicians, and independent cohorts.

### Limitations

First, the included studies were methodologically heterogeneous. As this was a scoping review, we did not perform a meta-analysis or formal study-level evidence grading; the findings therefore map the available evidence but do not provide pooled effect estimates or ranked certainty of evidence. Second, the search was restricted to English-language full-text studies indexed in PubMed, Embase, and the Web of Science Core Collection and did not include a separate grey-literature search; relevant evidence outside these sources may therefore have been missed. Third, the deliberate focus on modern generative LLMs does not cover the broader spectrum of AI methods used in epilepsy, and the conclusions should be interpreted as a snapshot of evidence available up to May 2026.

## Conclusions

Across 24 included studies, generative LLMs showed early, task-specific feasibility in epilepsy-related applications, particularly for processing unstructured clinical text and supporting documentation or knowledge-oriented tasks. The evidence was methodologically heterogeneous, largely retrospective or simulated, and insufficient to support high-stakes autonomous use.

The main barriers across studies were opaque reasoning, reliance on static or biased training data, limited local validation, privacy constraints, and unclear accountability when outputs influence care. General-purpose LLMs should not be used for epilepsy diagnosis or treatment decisions without clinician review. More defensible near-term uses include drafting, extraction, summarization, and research support with source checking and explicit clinician sign-off.

Future studies should develop task-specific, domain-grounded systems using high-quality epilepsy data and evaluate them with transparent protocols that include clinical oversight and verifiability. With these safeguards, LLMs may improve documentation, text review, and research workflows, but claims of direct clinical benefit require stronger real-world validation.

## Data Availability

No datasets were generated or analysed during the current study.

## Abbreviations

AUC: Area Under the Receiver Operating Characteristic Curve
EEG: Electroencephalography
EHRs: Electronic Health Records
ES: Epileptic Seizures
FS: Functional Seizures
GPT: Generative Pre-trained Transformer
ILAE: International League Against Epilepsy
LLMs: Large Language Models
NLP: Natural Language Processing
PRISMA-ScR: Preferred Reporting Items for Systematic reviews and Meta-Analyses extension for Scoping Reviews
RAG: Retrieval-Augmented Generation
